# The Multiple Facets of Iron Recycling

**DOI:** 10.3390/genes12091364

**Published:** 2021-08-30

**Authors:** Patryk Slusarczyk, Katarzyna Mleczko-Sanecka

**Affiliations:** Laboratory of Iron Homeostasis, International Institute of Molecular and Cell Biology, 02-109 Warsaw, Poland; patryk.slusarczyk@iimcb.gov.pl

**Keywords:** iron homeostasis, hemolysis, heme, hemoglobin, ferroportin, hepcidin

## Abstract

The production of around 2.5 million red blood cells (RBCs) per second in erythropoiesis is one of the most intense activities in the body. It continuously consumes large amounts of iron, approximately 80% of which is recycled from aged erythrocytes. Therefore, similar to the “making”, the “breaking” of red blood cells is also very rapid and represents one of the key processes in mammalian physiology. Under steady-state conditions, this important task is accomplished by specialized macrophages, mostly liver Kupffer cells (KCs) and splenic red pulp macrophages (RPMs). It relies to a large extent on the engulfment of red blood cells via so-called erythrophagocytosis. Surprisingly, we still understand little about the mechanistic details of the removal and processing of red blood cells by these specialized macrophages. We have only started to uncover the signaling pathways that imprint their identity, control their functions and enable their plasticity. Recent findings also identify other myeloid cell types capable of red blood cell removal and establish reciprocal cross-talk between the intensity of erythrophagocytosis and other cellular activities. Here, we aimed to review the multiple and emerging facets of iron recycling to illustrate how this exciting field of study is currently expanding.

## 1. Introduction

**Figure 1:**
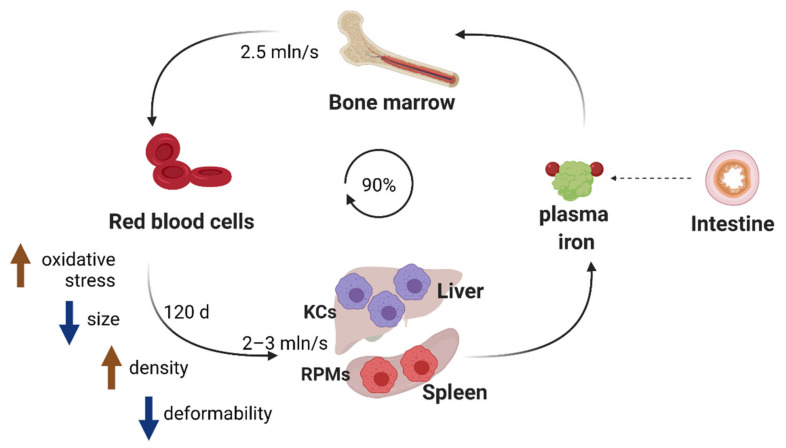
Iron recycling ensures the turnover of the body iron pool. Approximately 90% of iron needs for erythropoiesis are met by internal iron recycling from aged red blood cells. This task is accomplished by macrophages, predominantly Kupffer cells (KCs) in the liver and red pulp macrophages (RPMs) in the spleen. When erythrocytes age (in approximately 120 days in humans), their elasticity is reduced, which mediates their trapping in iron-recycling organs and further engulfment by KCs and RPMs.

Red blood cells (RBCs) or erythrocytes represent the most abundant cells in the human body, highly specialized for the shuffling of oxygen and carbon dioxide between the lungs and tissues. The binding of these gaseous particles is mediated by heme, an iron-containing prosthetic group that constitutes an integral part of the protein hemoglobin. The production of RBCs in a process called erythropoiesis is very rapid and yields approximately 200 billion RBCs daily, corresponding to 2.5 million every second [1] (Figure 1). This process tightly synchronizes intensive iron acquisition and the synthesis of heme with the translation of the globin polypeptides [2], which ultimately assemble stoichiometrically into hemoglobin composed of 2 α and 2 β globin chains, each biding one heme moiety. To perform their functions, mature RBCs are packed with remarkable amounts of hemoglobin that make up approximately one third of all erythrocytic proteins [3]. This is possible due to the expulsion of the nucleus and the elimination of all other key organelles, such as mitochondria, ribosomes, endoplasmic reticulum and the Golgi apparatus, throughout the erythroblast terminal differentiation [4]. 

The unique oxygen-carrying capacity of RBCs renders them particularly sensitive to oxidative damage. A fraction of hemoglobin-bound oxygen generates the superoxide anion that may further decompose into hydrogen peroxide and the highly reactive hydroxyl radical (OH) [5,6]. Hence, RBCs are well equipped with enzymes that protect them from reactive oxygen species (ROS), such as superoxide dismutase and catalyze, but their activity decreases during the lifespan of RBCs [7]. Recent studies demonstrated that RBCs contain also significant amounts of non-heme iron that need to be exported by the sole iron transporter ferroportin (FPN) to sustain proper RBC functions [8]. Genetic abrogation of this efflux pathway provokes oxidative stress and results in enhanced hemolysis. Therefore, the natural aging of circulating RBCs is in part underlain by progressive oxidative damage of proteins and lipids, which cannot be compensated due to the lack of organelles and cellular machinery responsible for de novo biosynthetic processes [5,6] (Figure 1). It has been estimated that the lifespan of RBCs is around 115 days [9] or 120 ± 4 days [6] in healthy humans and approximately 40 days in mice [10] (Figure 1). After reaching natural senescence or upon damage, RBCs are removed from the circulation by reticuloendothelial macrophages in a process called erythrophagocytosis. The continuous erythropoietic activity requires 2 × 10^15^ iron atoms every second and approximately 25 mg iron daily [11]. Since dietary iron availability is limited, mammals have evolved an efficient strategy for iron recycling, where approximately 90% of the iron demand for heme synthesis during erythropoiesis is ensured by the retrieval of iron from engulfed erythrocytes [12] (Figure 1). Hence, similarly to the production of RBCs, the rate of their sequestration is also estimated to be very high, reaching 2–3 million per second [13]. Over a human lifespan of around 60 years, it removes the mass of naturally aged RBCs that equals around 400 kg [6] and it continuously captures injured RBCs that otherwise could disrupt inside blood vessels and cause danger to surrounding tissues. Therefore, RBC clearance is a fundamental process for mammalian physiology that enables the turnover of the internal body iron pool. Nevertheless, the exact molecular mechanisms that are involved in the sequestration of senescent or damaged RBCs by distinct macrophage populations, primarily in the spleen and liver [14], are far from being fully elucidated. This is exemplified by a new provocative study that proposes a significant contribution of local hemolysis of RBCs in the spleen to their clearance. Furthermore, we have recently started to uncover signaling pathways important for the differentiation and biological roles of iron-recycling macrophages within their tissue microenvironment. Recent reports also illustrate how different pathophysiological conditions, such as recovery from acute anemia or inflammation, exploit the plasticity of iron-recycling macrophages to alter their functions or mediate the differentiation of novel subtypes of erythrophagocytic cells, distinct from those present under steady-state conditions. Lastly, new insights now emerge into the consequences of intensified RBC clearance for macrophage immune polarization. Within this review, we intend to cover all the above multiple facets of iron recycling. We will provide an update on the molecular mechanisms involved in the recognition and the removal of red blood cells, summarize insights into cues that mediate the development of iron-recycling cells and discuss reciprocal cross-talk between erythrophagocytosis intensity and other cellular functions of macrophages.

## 2. Recognition of Aged or Damaged RBCs by Iron-Recycling Macrophages

Over their 120-day lifespan, human RBCs travel approximately 500 km through the blood vessels, including narrow capillaries [15]. This is possible due to the very high elasticity of these unique cells. Their deformability depends on several factors: (i) interactions between the so-called RBC cytoskeleton, composed of spectrin, actin and ankyrin, with other integral membrane proteins, such as the 4.1 and 4.2 proteins and the cytosolic domain of the highly abundant Band 3 protein, (ii) proper ion and water homeostasis, (iii) optimal volume-to-surface ratio and (iv) membrane fluidity [16,17]. One of the major characteristic changes in aged RBCs is the loss of their elasticity, tightly linked to their dehydration, densification and shrinkage [13] (Figure 1). This is underlain by a few mechanisms. First, constant exposure of RBCs to oxidative stress leads to the oxidation and denaturation of hemoglobin, as well to the formation of lipid peroxides, which both may promote the clustering of Band 3 protein [12,18]. This, in turn, disrupts the erythrocytic membrane architecture, increasing cell rigidity. Second, the depletion of ATP levels [19] in aging RBCs and the decreased number of sodium pumps per cell [20] lead to the impairment of the active ion transport, thus causing a decline in the trans-membrane Na^+^ and K^+^ gradient [21]. Interestingly, although these alterations would be expected to mediate cell swelling, senescent RBCs lose water, becoming smaller and denser. This phenomenon may be linked to two players, the mechanoreceptor PIEZO1, whose activating mutation causes cause severe RBC dehydration, which is a hallmarks of dehydrated hereditary stomatocytosis [22], and the Gardos Ca^2+^ calcium channel [21]. One of the models proposes that normal circulatory shear stress activates PIEZO1 and elicits surges in cell calcium, which in turn stimulates the Gardos channel and leads to progressive losses of potassium and fluid. This model is consistent with the observation that naturally aged RBCs contain higher intracellular calcium levels [23]. Densification of naturally aging RBCs is associated with a modest depletion of hemoglobin that occurs mainly via the shedding of hemoglobin-containing vesicles that are cleared by the spleen and liver macrophages [24]. The formation of such vesicles is also responsible for a drop in RBC lipid content [5]. Importantly, the loss of RBC elasticity is not only characteristic of naturally aged RBCs but also a hallmark of defective or injured RBCs in genetic disorders including sickle cell disease, thalassemia and hereditary spherocytosis, as well as acquired pathologies such as sepsis, malaria or diabetes [25,26]. 

Experimental data showed that poorly deformable, aged or damaged erythrocytes are engulfed primarily in the liver and in the spleen [27] (Figure 1). The spleen is characterized by an open blood system and unique architecture that confers quality control for stiffened aged RBCs [25,28]. Within the red pulp of the spleen (which constitutes approximately 75% of the spleen mass), blood arrives into areas of reticular connective tissue that forms so-called splenic cords. To re-enter into the circulation, RBCs need to pass through narrow endothelial slits of the red pulp venous sinusoids. Those that are too rigid are retained within the spleen cords and hence can be recognized and phagocytosed by iron-recycling red pulp macrophages (RPMs), which constitute approximately 50% of the red pulp [25] (Figure 1). Quantitatively, the “open” blood flow pathway receives 10% to 20% of the splenic blood flow, and the biomechanical integrity of each RBC is thus verified by the spleen approximately every 2 h [25].

It is less clear if and how senescent and poorly deformable RBCs are trapped in the liver. One report suggested that liver sinusoidal endothelial cells (LSECs) aid in the tethering of aged RBCs within hepatic sinusoids, thus facilitating their engulfment by liver macrophages called Kupffer cells (KCs) [29] (Figure 1). Recent in-depth imaging studies of the hepatic tissue architecture also illustrate that KCs not only reside within the sinusoidal vessels, often occupying most of their lumen, but also spread into the space of Disse, a niche between LSECs and hepatocytes [30]. It is plausible that such a local microenvironment, with the narrow lumen of sinusoids lined to LSECs and packed with KCs that protrude across the endothelial wall, is involved in capturing rigid RBCs. 

As illustrated by the experimental data, macrophages are the major cell type that sequesters RBCs in the steady state. The liver is characterized by the highest abundance of F4/80-positive macrophages, classified typically as KCs [31]. Consistently, the liver sequesters the largest amounts of stressed RBCs [27]. Single-cell transcriptome data of liver non-parenchymal cells enriched for F4/80-positive cells revealed that KCs show some degree of heterogeneity, comprising a few distinct clusters [32]. Indicated by the presence of RBC-derived mRNAs, only a small subset might be active in RBC engulfment in a given moment. Youssef et al. demonstrated that in the spleen, mostly RPMs, and, to a much smaller extent, Ly6C-high monocytes, can engulf senescent RBCs, but other cell types, such as granulocytes, dendritic cells or lymphocytes, exhibit very little or no erythrophagocytic capacity [33]. Bian et al. showed that RPMs are the most efficient phagocytes towards RBCs as compared with other splenic macrophage subsets, such as metallophilic or marginal zone macrophages [34]. Finally, in line with the observation that, at the systemic level, the bone marrow contributes minimally to the clearance of stressed RBCs [27], macrophages in this tissue were shown to be much less efficient in erythrophagocytosis than RPMs [33]. It is important to note that the clearance of stressed RBCs is rapid and takes 10–20 min [27] up to a few hours [33,35].

**Figure 2:**
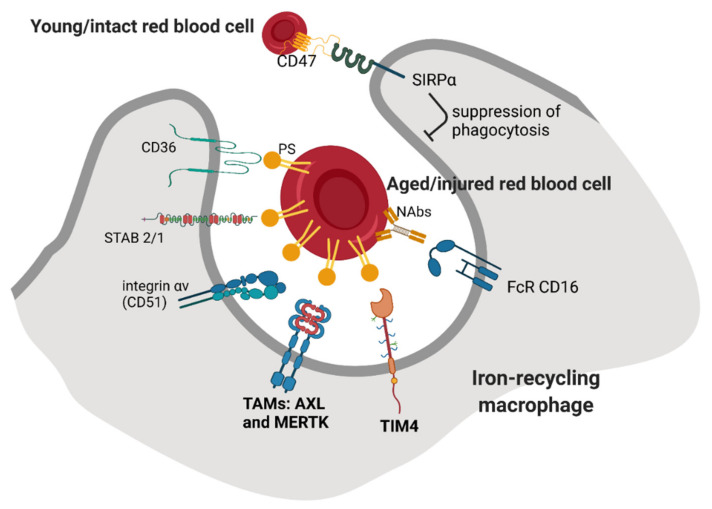
Recognition of red blood cells’ biochemical integrity by iron-recycling macrophages. Aged/injured erythrocytes expose exoplasmic phosphatidylserine (PS) on their surface and become opsonized by naturally occurring antibodies (NAbs). Among the cognate receptors for these senescence signals that are expressed on RPMs and KCs, TAM receptors AXL and MERTK, TIM4 and Fc receptor CD16 are the most abundant. A “don’t eat me” signal that prevents the clearance of young and intact erythrocytes is provided by the interaction between SIRPα at the surface of the macrophage with CD47 expressed by erythrocytes.

Recognition and phagocytosis of “trapped” RBCs by macrophages involve additional signals, but it is not well understood if these mechanisms fully overlap between KCs and RPMs. One of the proposed recognition modes involves the binding of phosphatidylserine (PS), a phospholipid that is typically exposed on the external leaflet of the plasma membrane of apoptotic cells [5] (Figure 2). It has been shown that forced exposure of a PS derivative at the surface of RBCs triggers their clearance, predominantly in the spleen [36]. Furthermore, aged human RBCs isolated from the blood according to their high density [37] or mouse RBCs that remained in the circulation for a prolonged time (marked by prior biotinylation) [38] exposed PS on the surface and could be efficiently cleared when transfused into mice. However, other studies reported that the PS externalization does not correlate with natural RBC aging, but rather is a hallmark of injured and stressed RBCs that need to be sequestered [39]. Consistently, RBCs isolated from sickle cell disease or thalassemia patients that are prone to premature clearance contain a higher percentage of cells positive for exoplasmic PS than RBCs from control subjects [40,41]. Moreover, others showed that the induction of oxidative stress by chemical agents that mimic ROS buildup during physiological RBC aging does not lead to PS exposure [42]. In sum, PS exposure is a hallmark of defective or injured RBCs in certain pathophysiological conditions and might contribute to the clearance of naturally senescent RBCs, but it likely does not act as the most critical signal for their removal. Nevertheless, published RNA sequencing data show that both KCs [43] and RPMs [44] express relatively high levels of scavenger receptors that recognize PS (Figure 2). These include mainly TIM4, a marker of native tissue-resident macrophages [45], and the TAM receptors AXL and MERTK, whose high levels on KCs were also detected using liver tissue immunofluorescence [46]. In the context of TAM receptors, they act via an additional ligand GAS6, but its exact cellular source for the recognition of aged or injured RBCs would need to be specified. Alternatively, PS can be also bound by another ligand, MFG-E8, and further recognized by α_V_β_III_ integrin [47]. The gene *Itgav* that encodes for integrin α_V_ (CD51) is expressed by both KCs and RPMs, albeit in low levels. Other receptors that may recognize PS are stabilins STAB1 and STAB2. They are only mildly expressed by KCs but are abundant in the liver LSECs, and they were implicated in the aforementioned tethering of stressed RBCs within liver sinusoids [29]. In RPMs, STAB2 shows an intermediate level of expression as compared to other receptors, and STAB1 is almost absent. The extent to which stabilin-mediated sequestration may operate within the splenic sinusoids and whether it can involve endothelial cells is still unknown. Another important scavenger receptor for PS, CD36, was implicated mainly in the RBC removal in malaria, which affects both infected and non-parasitized RBCs [48,49]. This is supported by the observation that individuals carrying a CD36 nonsense mutation are protected from malaria-induced anemia that was linked to augmented erythrophagocytosis [50]. CD36 appears to be weakly expressed by RPMs, whereas in KCs, depending on the dataset, the expression is either low or high [43]. Other receptors that recognize PS and were described in the clearance of apoptotic cells, such as BAI-1, or TIM1 and 3 [51,52,53], show little or no expression in iron-recycling cell macrophages from the liver and spleen, whereas SR-AI (encoded by *Msr1*) has intermediate expression levels in KCs and low in RPMs.

Another important mechanism that was proposed for the recognition of senescent RBCs is mediated by their opsonization by naturally occurring antibodies (NAbs) [6] (Figure 2). It was shown that those physiologically senescent RBCs that either constituted the densest fraction from collected human RBCs [54,55], circulated in the blood of dogs for at least 100 days [56] or were enriched in mice due to the hypertransfusion protocol [57] all showed increased binding of autologous antibodies on their surfaces. The majority of them were of the IgG isotype, but some were IgMs and IgAs [54,55]. Further studies have indicated that damaged or stressed RBCs, such as those exposed to oxidative agents or present in sickle cell patients, are also opsonized by NAbs [58,59]. The main mechanism responsible for the formation of antigens on the RBCs’ surfaces is the interaction between denatured hemoglobin (termed hemichrome) with the Band 3 protein. This disrupts membrane structure and leads to Band 3 clustering and the formation of protein aggregates that also contain other RBC components [59,60]. Consistently, according to RNA sequencing data, both RPMs and KCs express high levels of the Fc receptors CD16 but very little CD32 (Figure 2). It was also shown that the binding of autologous antibodies promotes further opsonization by complement components, thus strengthening the phagocytic removal [6,61]. However, the complement receptors CR1/CR2 (CD35/CD21), CR3 (*Itgam*), CR4 (*Itgax*) or CD88 are very low or absent in RPMs and KCs, raising the question of whether physiologically complement opsonization indeed facilitates erythrophagocytosis.

Some other signals for the recognition of senescent RBC were proposed and involve the loss of sialic acid [6] and a functionally related interaction between adhesion molecules of RBCs (Lu/BCAM) and the extracellular matrix component laminin-α5 [62]. Nevertheless, opsonization by NAbs as well as PS exposure emerge as dominating senescence signals. Interestingly, however, the genetic loss-of-function studies of their cognate receptors in the context of erythrophagocytosis in vivo are still lacking. Therefore, the exact molecular nature of such “eat me” interactions between senescent or stressed RBC and endogenous RPMs and KCs, as well as their precise contribution to iron recycling both in the liver and the spleen, remain elusive. However, it should also be stated that such studies may be challenging as the iron-recycling capacity in the liver and the spleen exhibits some plasticity and may be supported by recruited monocytes, distinct from native RPMs and KCs (as described in detail below). Furthermore, one study proposed that natural aging as opposed to in vitro stress induces relatively moderate senescence signals that likely act additively and promote the removal of RBCs in vivo but not in vitro [38]. This raises also the possibility that the tissue microenvironment promotes RBCs’ engulfment, which will be discussed below. 

In contrast to the signals that promote erythrophagocytosis, which remain not completely deciphered, it is well established that a “don’t eat me” signal is provided by an interaction between CD47 on the surface of RBCs with the SIRPα receptor on macrophages (Figure 2). It was shown that this axis prevents phagocytosis of undamaged RBCs and protects also other cell types and platelets [34]. Upon transfusion, CD47-null RBCs are rapidly sequestered from the circulation. Strikingly, this clearance is exclusively accomplished by the splenic, not liver, macrophages, and does not depend on NAbs or complement opsonization [63]. However, the binding of CD47 by its receptor is effective in suppressing antibody-mediated RBC recognition but fails to prevent the uptake of oxidatively stressed RBCs [64]. It was also demonstrated that the high degree of RBC rigidity overrides the “self” signaling conferred by CD47 [65]. It has been proposed that a drop in CD47 levels accompanies the physiological aging of murine RBCs and contributes to their natural turnover [38,66]. Decreased levels of CD47 may also represent one of the clearance signals of RBCs that are stored for a prolonged time before transfusion [67]. Mechanistically, the inhibition of phagocytosis upon CD47–SIRPα interaction depends on the binding of the phosphatases SHP-1/2 to the cytoplasmic domain of SIRPα [68]. One important study proposed that CD47 may also undergo conformational alteration during RBC aging and thus switch from an inhibitory to stimulatory signal for phagocytosis [69]. This mechanism may involve the binding of thrombospondin-1 to CD47 and other domains on the cytoplasmic part of SIRPα than those required for the inhibition of phagocytosis. Interestingly, since cancer cells often induce CD47 to evade immune eradication [70] and malaria parasites infect young CD47-high RBCs to avoid clearance, targeting of the CD47–SIPRα axis is of high therapeutic interest [71,72].

Lastly, it is worth mentioning that RPMs in the spleen may play an important role in maintaining RBCs’ fitness. It was shown that RBCs isolated from splenectomized patients contain cytoplasmic inclusion bodies (packed with chromatin, denatured hemoglobin or excessive iron), thus suggesting that the spleen facilitates their clearance [73].

## 3. Sequestration of Hemolytic Erythrocyte Components

**Figure 3:**
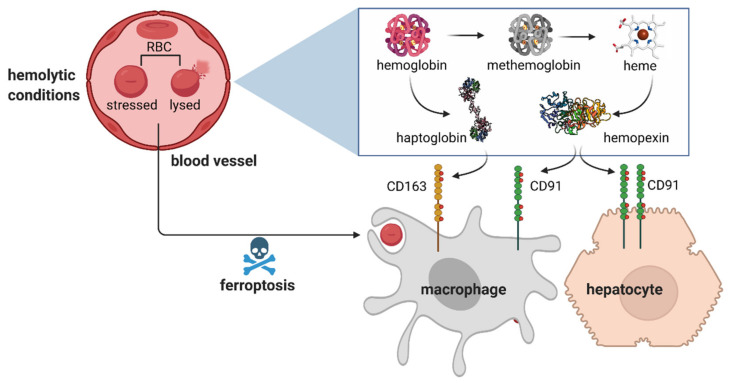
Sequestration of hemolytic erythrocyte components. During hemolysis, the components of RBCs, free hemoglobin and heme, are sequestered by the plasma scavenging proteins, haptoglobin and hemopexin, respectively. The removal of the formed complexes by macrophages and hepatocytes is mediated via the CD163 and CD91 receptors. Under the conditions of erythrolytic stress, macrophages are depleted by intensified erythrophagocytosis that leads to ferroptosis.

Early kinetic studies implied that in the steady state in humans, slightly above 10% of RBCs may undergo intravascular hemolysis, thus releasing free hemoglobin (Hb) [74] (Figure 3). It is well established that the increased prevalence of hemolytic events is a hallmark of several hereditary anemias, including sickle cell disease, spherocytosis, autoimmune hemolytic anemia, erythropoietic protoporphyria and pyruvate kinase deficiency [75,76]. Free Hb is sequestered by haptoglobin, an acute phase plasma protein that is primarily produced in the liver [77]. The complex of Hb–haptoglobin is taken up via CD163-mediated endocytosis [78] (Figure 3). Both KCs and RPMs express high levels of CD163, as indicated by sequencing data [43,44] and immune detection in tissue sections [79,80]. Studies using radiolabeled Hb show that the clearance of injected hemoglobin is rapid [81] and mostly accomplished by the liver, spleen and kidney, with their contributions varying depending on the Hb dose [81,82]. Interestingly, pharmacokinetic experiments in non-rodent species show that the clearance rate for the Hb–haptoglobin complex is much slower than for free Hb [83] and, consistently, some reports implied that other routes for Hb sequestration exist that are independent of haptoglobin and/or CD163 [84,85]. One such mechanism involves renal glomerular filtration, as indicated by profound renal Hb uptake and iron loading in the kidney of haptoglobin-null mice [81,82]. However, it remains to be established if other means of alternative Hb clearance exist in the body. This is a particularly important question as the haptoglobin pool in the serum may become depleted during a prolonged hemolytic crisis [76].

Under prooxidative conditions, ferrous iron of hemoglobin may undergo oxidation [86]. This leads to the formation of methemoglobin, which is unstable and releases free heme (Figure 3). Free heme is sequestered by another scavenging protein, hemopexin, which protects from heme-induced vascular dysfunction and heme-triggered inflammation [87,88]. The complex of heme–hemopexin is bound by the receptor CD91 (LRP-1) [89], which is expressed mainly on hepatocytes and, to a lesser extent, on iron-recycling macrophages [43,44] (Figure 3). This is also in agreement with the fact that mice deficient in heme catabolism that consequently lose macrophages due to heme-mediated toxicity show iron re-distribution to hepatocytes [90].

## 4. Hemolysis-Driven Iron Recycling Model

In light of the well-established view that the removal of naturally aged or stressed RBCs is accomplished predominantly via phagocytosis, a recent study by Klei et al. proposed a new mechanism mediated by local hemolysis in the spleen [80]. It was demonstrated that human spleen tissue contains a proportion of RBCs devoid of hemoglobin, so-called erythrocyte ghosts. In mice, representative flow cytometric data implied that a subset of red pulp macrophages phagocytosed preferably such ghosts upon transfusion of senescent RBCs. In support of the proposed model, in vitro flow assay and state-of-the-art imaging of human spleen tissue indicated that the hemolysis of aged erythrocytes is driven by the interaction between laminin-α5 located in sinusoids with the Lu/BCAM adhesion complex at the surfaces of RBCs. The authors further propose that hemoglobin is locally sequestered by splenic haptoglobin and the complex undergoes endocytosis via CD163 expressed by RPMs. Although this novel model is highly interesting and undoubtedly contributes to the RBC turnover, it raises additional questions that may be addressed by further investigations. For example, it would be informative to quantify precisely how much hemolysis-driven vs. phagocytosis-driven RBC removal contributes to the overall turnover of RBCs and if any hemolytic events also accompany erythrocyte sequestration in the liver. Another important question would be if indeed CD163-mediated Hb uptake by macrophages represents the sole mechanism for Hb uptake, taking into account that other haptoglobin or/and CD163-independent mechanisms may exist. The authors of this novel report also used the gating strategy for RPMs, contrary to other laboratories that investigated the functions of these cells [33,91,92,93,94] (based on their autofluorescence rather than surface markers). Klei et. al did not discuss their intriguing data in relation to the work of Youssef et al., where senescent RBCs that contained GFP in their cytoplasm were employed for transfusion [33]. In their hands, approximately 40% of RPMs were GFP-positive 2 h post-injection, indicating classical phagocytic uptake, and already after 5 h, RPMs induced the heme-responsive gene *Spi-c*, suggesting that RBCs were efficiently degraded and heme was released inside the cells. Furthermore, another report by Ma et al. demonstrates that mice with a macrophage-specific activating mutation of the PIEZO1 calcium channel show increased phagocytic capacity of RPMs, thus driving intensified RBC removal. How these important data could be interpreted in light of the hemolysis-driven RBC sequestration remains to be better explained. In summary, the novel and provocative data presented by Klei et al. would benefit from further follow-up studies.

## 5. Erythrophagocytosis and Intracellular Iron Handling in Iron-Recycling Cells 

**Figure 4:**
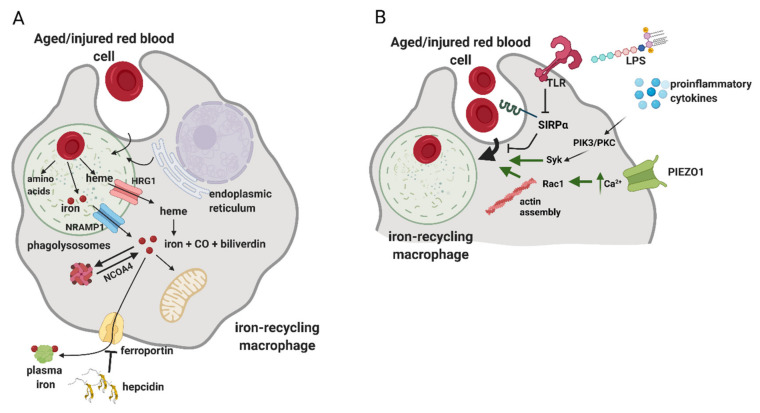
The process of erythrophagocytosis and the emerging mechanisms of its regulation. (**A**) RPMs and KCs are proficient in recognizing and engulfing aged RBCs, which, to a large extent, is mediated by the process called erythrophagocytosis. Upon the formation of phagolysosomes, cellular components of RBCs are degraded, globins are hydrolyzed to amino acids and heme is released. Non-heme iron that is present in RBCs is transported by NRAMP1. Heme is transported to the cytoplasm by HRG1 and subsequently catabolized by heme oxygenase 1 (HO-1) to carbon monoxide (CO), biliverdin and ferrous iron. Iron is sequestered by ferritin and, upon increased iron demand, can be released from ferritin via ferritinophagy in an NCOA4-dependent fashion. Iron efflux occurs via ferroportin and replenishes the pool of plasma iron. The process of iron release from macrophages is tightly regulated by hepcidin, a small liver-derived hormone that mediates ferroportin degradation and/or occlusion, hence preventing iron release from the macrophage iron reservoir. (**B**) Recent advances showed that the intensity of erythrophagocytosis can be enhanced by proinflammatory conditions and bacterial components, such as lipopolysaccharide (LPS). These effects are mediated by the downregulation of SIRPα and PIK3/PKC/Syk-dependent signaling. The PIEZO1 mechanoreceptor was identified as a positive regulator of erythrophagocytosis and was shown to control calcium levels and actin remodeling in iron-recycling macrophages.

Mechanistic details of how phagocytosis of RBCs is accomplished and regulated are still incompletely understood (Figure 4). Studies in cultured macrophages implied that this process may involve components of the autophagy machinery, hence engaging a route of so-called LC3-associated phagocytosis (LAP) [95]. LAP is known as means of the removal of apoptotic cells that triggers the anti-inflammatory and immunosuppressive responses of macrophages, a phenomenon that is of high clinical interest in the context of anti-tumor immunity [96]. However, loss-of-function studies that would address whether LAP-deficient RPMs or KCs in vivo exhibit defects in erythrophagocytosis are still lacking. As illustrated by in vitro microscopy imaging of erythrophagocytosis in primary macrophages, upon the formation of phagosomes, LAMP-1-positive lysosomes are recruited, thus maturating into phagolysosomes [97] (Figure 4A). Interestingly, this process also might be supported by the fusion with endoplasmic reticulum membranes [97,98]. Cellular components of RBCs are degraded, globins are hydrolyzed and heme is released. Both in vitro and in vivo data demonstrate that the decomposition of RBCs is a relatively rapid process, as already within the few first hours post-erythrophagocytosis, heme-responsive genes are induced [33,99,100,101]. Since RBCs contain both non-heme and heme iron [8], transporters for both these cargoes are recruited to phagolysosomal membranes (Figure 4A). According to in vitro data obtained in cultured primary macrophages, the former include primarily the metal transporter NRAMP1 [97]. Mice deficient for NRAMP1 show enhanced iron retention in the spleen and liver, supporting the physiological role of NRAMP1 in iron recycling from RBCs [102]. The main heme-catabolizing enzyme, heme oxygenase 1 (HO-1), was shown to reside primarily in the cytoplasm of macrophages [97]. Consistently, the heme transporter HRG1, originally identified in genetic screens in *C. elegans* [103], was shown to be recruited to erythrophagolysosomes to enable heme delivery to the cytoplasm [3,97]. Further studies using zebrafish and mouse models further corroborated the critical role of HRG1 in iron recycling, especially by demonstrating that HRG-1 deficiency is lethal in mice fed an iron-deficient diet [94,104]. Pek et al. also demonstrated that the lack of HRG1 in mice leads to the formation of hemozoin, the heme aggregate previously found only in parasites, within the enlarged lysosomes of iron-recycling macrophages [94].

In the cytoplasm, HO-1 degrades heme to carbon monoxide, biliverdin and ferrous iron (Figure 4A). Lack of HO-1 leads to a severe phenotype in mice, hallmarked by embryonic lethality of approximately 90% of homozygous knock-out mice [90,105]. Those HO-1-null mice that survive show a progressive loss of KCs and RPMs due to heme-driven toxicity and develop fibrosis in the red pulp of the spleen. Nine cases of human mutations in *HMOX-1* encoding for HO-1 were reported in the literature and the majority of these patients were characterized with chronic inflammation, hemolysis and asplenia, thus further corroborating the critical roles of HO-1 for maintaining blood homeostasis. Ferrous iron generated by HO-1 replenishes the cytoplasmic reservoir of metabolically available iron, called the labile iron pool (LIP) [11] (Figure 4A). Iron from LIP is sequestered by ferritin, a nanocage heteropolymer that oxidizes and stores iron, which is also post-transcriptionally induced upon RBC sequestration [99]. To the best of our knowledge, the characterization of iron-recycling capacity and body iron indices in macrophage-specific ferritin-null mice is still lacking. Under conditions of increased iron demand, ferritin is targeted by nuclear receptor coactivator 4 (NCOA4) for autophagic degradation, called ferritinophagy [106]. A proportion of iron from LIP is released by iron-recycling macrophages by the sole iron exporter FPN [107], which itself is induced following erythrophagocytosis [100,101] (Figure 4A). Iron efflux from macrophages is coupled with the oxidation of ferrous to ferric iron by ceruloplasmin and replenishes the pool of serum iron required for continuous erythropoiesis [11]. Hence, mice with macrophage-specific deletion of *Slc40a1* that encodes for FPN are presented with mild anemia accompanied by splenic and hepatic iron accumulation, phenotypes that are exacerbated by hemolytic challenge or iron-deficient diet [108]. The process of iron release from RPMs is tightly regulated by hepcidin, a small liver-derived hormone that adjusts plasma iron levels to body iron needs [107,109]. Hepcidin acts by mediating FPN degradation and/or occlusion, thus causing macrophage iron retention and serum hypoferremia [109,110]. This is characteristic of the inflammatory conditions that typically lead to high hepcidin levels [111], but hepcidin-independent and rapid FPN downregulation in response to pathogen-associated molecules was also described [112]. Conversely, low hepcidin levels, which are a hallmark of iron deficiency and other conditions characterized with high iron demand for erythropoiesis, lead to FPN stabilization and boosted iron export capacity of iron-recycling macrophages [11,113,114]. Despite the growing body of knowledge, we still do not understand completely how iron is trafficked in iron-recycling macrophages and how iron from LIP is distributed between intrinsic metabolic cellular needs and the export route.

Regarding the fate of heme iron following endocytosis of the Hb–haptoglobin complexes, another heme transporter called HCP1 was implicated in the delivery of heme to the cytoplasm from the endosomal compartment [115]. It remains to be better understood if other mechanistic details regarding heme and iron processing upon Hb uptake may reflect those associated with erythrophagocytosis.

## 6. Regulation of the Erythrophagocytosis Rate

The processes of RBC phagocytosis and digestion are considered largely constitutive. However, some reported data indicate that erythrophagocytosis may be subjected to different regulatory mechanisms (Figure 4B). Delaby et al. found that primary macrophages treated with proinflammatory stimuli, lipopolysaccharide (LPS) and interferon γ (INFγ) show increased phagocytic capacity towards RBCs [100]. In vivo, Bian et al. demonstrated that treatment of mice deficient for the SIRPα–CD47 inhibitory axis with interleukin 17 potentiates the erythrophagocytic activity of RPMs. In vitro follow-up studies implied that this response is mediated by protein kinase C and kinase Syk and is calcium-dependent. Another more recent study elegantly showed that the induction of a strong inflammatory response in mice leads to the downregulation of SIRPα in a toll-like receptor (TLR)-dependent fashion and, in parallel, enhances the phagocytic capacity of RPMs via PI3K- and Syk-dependent signaling [93]. Consistent with the role of calcium in controlling phagocytic activity, it was recently shown that PIEZO1, a mechanically activated nonselective cation channel, is an important regulator of erythrophagocytosis [116]. It was found that mice carrying a macrophage-specific activating mutation of PIEZO1 show enhanced phagocytic capacity of splenic RPMs. Mechanistic studies showed that this is driven by increased calcium signaling and the activation of small G protein Rac1, which controls actin cytoskeleton remodeling. In this context, it is interesting to note that heme accumulation in cultured primary macrophages was reported to inhibit phagocytosis of non-RBC-related cargoes via activation of small G protein Cdc42, which, similarly to Rac1, promotes actin polymerization. The observation that both suppression and excessive activity of small G proteins impairs phagocytosis may be explained by the fact that the complete engulfment of large cargoes relies on both actin assembly and the subsequent rapid deactivation of G proteins and actin disassembly [117]. Finally, another recent work identified transient receptor potential melastatin 7 (TRPM7), a cation channel with kinase activity, as a suppressor of RPMs’ phagocytic activity through a mechanism that likely involves cytosolic alkalinization [118].

## 7. Development and Plasticity of Iron-Recycling Macrophages

**Figure 5:**
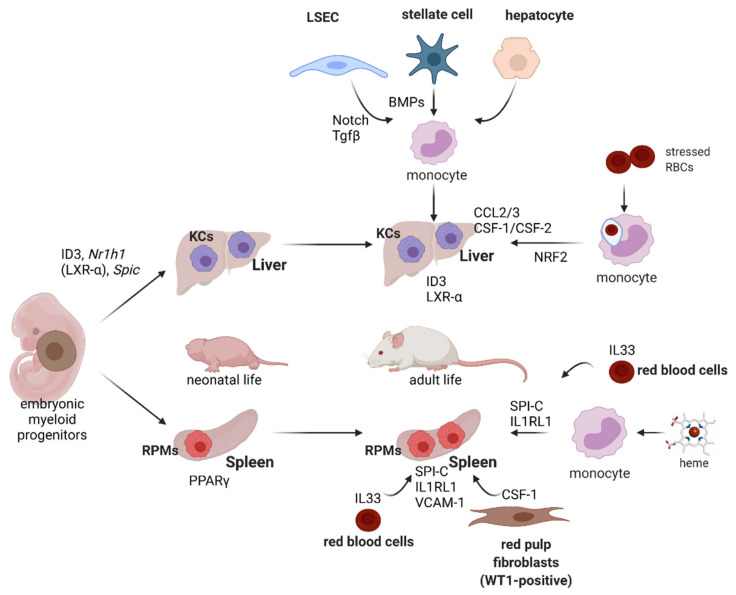
Pathways that imprint KCs’ and RPMs’ identity and enable plasticity of iron-recycling cells. RPMs’ neonatal expansion depends on PPARγ, whereas in adult life, their identity and numbers are maintained by SPI-C, VCAM-1, the IL33–IL1RL1 axis and CSF1 released by red pulp fibroblasts. Upon hemolytic stress, differentiation of monocytes into RPM-like cells is mediated by *Spi-c* induction due to heme loading and IL33 signaling. KC development is controlled mainly by ID3 and LXR-α. Differentiation of monocytes into functional KCs in emptied KC niche requires interaction with liver sinusoidal endothelial cells (LSECs), hepatic stellate cells and hepatocytes and soluble factors that are secreted by these cells, including Notch ligands, TGF-β and BMPs. Under erythrolytic stress, blood monocytes were shown to engulf stressed RBCs and differentiate into KC-like cells, which is driven by the chemotactic factors present in the liver and intrinsic NRF2 signaling in monocytes.

RPMs and KCs belong to tissue-resident macrophages (ResMϕs), highly heterogeneous and multifunctional cells in the mammalian body that uniformly sustain homeostasis within specific microenvironments [119,120]. Genetic fate-mapping studies have shown that ResMϕs develop prenatally from embryonic progenitors, including yolk-sac macrophages and fetal liver monocytes [121,122] (Figure 5). ResMϕs from different organs show highly distinct transcriptional signatures and epigenetic landscapes [123,124,125], which reflect the niche-specific signaling programs that determine their identity. Differentiation of RPMs is governed by a few interconnected mechanisms that were discovered over the last few years (Figure 5). First, during early postnatal life, RPMs undergo rapid expansion that is accompanied by the induction of the peroxisome proliferator-activated receptor gamma (PPARγ) transcription factor [126]. Genetic deletion of this regulator using the *Vav1*-Cre line that is specific to hematopoietic cells, including differentiating fetal monocytes, intrinsically abolished neonatal RPM development [126]. The few PPARγ-deficient RPMs that remained showed differential transcriptomes to wild-type control cells, but the exact roles of the identified genes were not investigated in depth by the authors. One mechanistic detail provided in this work shows that PPARγ-null RPMs fail to downregulate CD88 and integrin CD11a at the protein levels, which is characteristic of developing wild-type RPMs. Interestingly, PPARγ-deficient RPMs, although much less in number, exhibited a similar capacity for erythrophagocytosis as wild-type cells. Finally, it was shown that the inducible deletion of PPARγ in adult mice has no effects on RPM numbers and only mildly alters their transcriptional signature. Another important transcription factor that is dispensable for neonatal RPM expansion [126] but is critical for their survival in adulthood is SPI-C [127]. SPI-C-null mice show near-total loss of RPMs in the spleen, but a normal representation of KCs and other immune cells. Another report showed that mice lacking the VCAM-1 receptor are likewise characterized by reduced RPM numbers [128]. Similar to the *Vav1*-Cre-driven PPARγ-null mice, SPI-C knock-out animals also surprisingly show normal hematological parameters and serum iron levels, but progressively accumulate iron in the spleen, indicative of defective iron recycling [127]. It may be speculated that the lack of iron-deficient phenotype in both models is due to the increased erythrophagocytic function of liver KCs or/and enhanced iron absorption from the diet. In line with these data, all three models (lacking PPARγ, SPI-C or VCAM-1 in RPMs) respond also to hemolytic stress similarly to wild-type mice. 

Further work identified *Spic* as the heme-responsive gene and has shown that induction of *Spic* is responsible for the differentiation of heme-loaded monocytes into RPMs under hemolytic stress [91] (Figure 5). Mechanistically, it was demonstrated that *Spic* transcription is repressed by BACH1, a factor that undergoes degradation in response to heme accumulation. This mechanism thus establishes a link between the major metabolite of RBCs’ and RPMs’ identity. Recent studies demonstrated that heme alone does not trigger the development of fully mature RPMs [92]. The final steps of the RPM differentiation process are dependent on the cytokine IL33, acting via its receptor IL1RL1 present on RPMs and the downstream signaling, via the ERK kinase and GATA1 transcription factor (Figure 5). Interestingly, IL-33 was shown to be derived from RBCs that recently were identified as an important source of various cytokines [129]. Finally, red pulp fibroblasts that express the WT1 transcription factor and release the CSF-1 cytokine were demonstrated to provide a meshwork that anchors and nourishes RPMs [130].

Niche-derived or cell-intrinsic signals that drive KCs’ development and are required for their homeostasis are beginning to be understood (Figure 5). Sequencing data of the subsequent differentiation steps of cells during organogenesis, from myeloid progenitors through so-called pre-macrophages to specialized macrophage populations, revealed that KCs’ identity is hallmarked by the induction of the *Id1*, *Id3*, *Nr1h3* and *Spic* genes [131]. Consistently, deletion of *Id3* (encoding for the transcriptional regulator inhibitor of DNA binding 3; ID3) or *Nr1h3* (encoding for the transcription factor liver X receptor-α; LXR-α) in mice led to the loss of KCs’ identity and depletion of this population [131,132]. Transcriptomic data generated by the Immgen Consortium [123] prompted studies that identified *Clec4f* as another highly KC-specific gene, although its exact role in KCs’ physiology remains elusive [133]. Nevertheless, this allowed for the targeted ablation of this macrophage population from mice using a diphtheria toxin receptor-mediated strategy [133]. Scott et al. further found that the replenishment of the emptied KC niche is accomplished by the recruitment of bone marrow monocytes. More recently, two important studies sought to decipher the mechanistic details that regulate KC niche repopulation [30,43] (Figure 5). They revealed that the recruited monocytes established close contacts with LSECs, hepatocytes and hepatic stellate cells (HSCs) in the liver sinusoids. LSECs were shown to provide Notch ligands, mainly DLL4, to induce LXR-α in monocytes, a response that was potentiated by BMP ligands released by HSCs and LSECs, with HSC-derived BMP9 playing likely a dominant role. Other factors identified as required for the imprinting of KCs’ identity on replenished monocytes included transforming growth factor-β (TGF-β) released by the LSECs and endogenous hepatic LXL ligands. Furthermore, contact with hepatocytes was identified as means to induce *Id3* expression by a yet unknown mechanism. Interestingly, KCs of monocyte origin that are recruited to the emptied niche are highly similar to embryonically derived KCs [133]. Twelve genes that were >1.5-fold higher in original resident KCs compared to monocyte-derived cells included TIM4, a marker of ResMϕs [45], Hb–haptoglobin receptor CD163 and BMP receptor BMPPR1, which might be linked to the role of BMPs in the de novo occupancy of the KC niche, as described above [30,43].

The ResMϕs of embryonic origin are proposed to maintain themselves due to their long-term self-renewal capacity, akin to that of stem cells [134]. The percentage of macrophages that are positive for Ki-67, a proliferation marker, is approximately 2% in KCs and 5–12% in the RPM population [33,133]. Interestingly, in both these niches, partial depletion of the resident macrophages leads to enhanced proliferation of the remaining cells that contribute to de novo niche occupancy [33,133]. However, the mechanisms that regulate this increased mitotic activity are not yet elucidated. 

Recent studies demonstrated that, under steady-state conditions, tissue macrophages of prenatal origin co-exist in several organs with those that are derived postnatally from monocytes [135]. By using a newly created reporter mouse model (driven by Cre recombinase under the monocyte-specific *Ms4a3* promoter) where cells of monocyte origin specifically expressed a fluorescent protein, it was revealed that in young (8-week-old) mice, approximately 20% of RPMs are already replenished from monocytes [135]. This is in agreement with the previous piece of work [133]. In contrast to RPMs, Liu et al. identified the minimal contribution of monocytes to the population of KCs in the steady state. This partially contradicts the findings of Scott et al. [133], who previously reported the replenishment rate of KCs to be approximately 30%. This discrepancy likely arises from the differential methodology, as the earlier study used an approach of adoptive transfer of congenic bone marrow cells into pups within the first few days after birth [133]. It remains an open question whether iron-recycling macrophages of monocyte vs. embryonic origin differ from each other functionally under steady-state conditions.

As exemplified above for KCs, replenishment from bone marrow-derived monocytes is particularly enhanced when the homeostasis of the resident macrophage population is disturbed. Early studies estimated that, physiologically, one KC engulfs approximately one erythrocyte per day [136]. For RPMs, around 10% of cells are actively phagocytosing in a given moment, and, typically, one erythrocyte can be detected in one red pulp macrophage [44]. KCs and RPMs are rapidly depleted by forced erythrophagocytosis [27,33], intravascular hemolysis or pharmacological inhibition of HO-1 [91]. This was proposed to occur due to heme cytotoxicity and ferroptosis, a form of cell death triggered by lipid peroxidation and promoted by the excessive cytoplasmic iron pool [137] (Figure 3). In the spleen, the differentiation of new monocyte-derived RPMs is mostly driven by the heme-mediated induction of *Spic* [91]. The extent to which the newly established RPMs resemble those originally residing in the niche, in terms of their transcriptional signature and iron-recycling functions, remains to be elucidated. Another important piece of work demonstrated that, upon exposure to an excess of stressed RBCs, and the depletion of resident iron-recycling macrophages, the liver, but not the spleen, takes over RBC clearance [27] (Figure 5). Damaged RBCs are first engulfed by circulating Ly6C-high monocytes, which migrate to the liver following chemotactic signals conferred by CCL2 and CCL3. Next, a high ratio of CSF-1/CSF-2 cytokines that is characteristic of the liver, but not the spleen, was shown to drive the further differentiation of monocytes into a myeloid population, which the authors termed transient macrophages (tMϕs). These cells, characterized by high FPN levels, but negative for the ResMϕs marker TIM4 [45], further acquired a transcriptional profile that resembled iron-recycling KCs, but still differed significantly from resident KCs of embryonic origin. This also implies that these KC-like cells that emerge after erythrolytic stress have a distinct identity from those that are recruited to the liver when the KC niche is depleted by the diphtheria toxin strategy [133]. The KC-like cells catabolized RBC-derived heme and delivered iron to hepatocytes. When the stress imposed by stressed RBCs declined, this population disappeared from the liver. The appearance of tMϕs was dependent on the external chemotactic cues described above and intrinsic signaling via NRF2 (Figure 5). Interestingly, similar responses hallmarked by monocyte-mediated niche replenishment were absent in the spleen, a phenomenon explained by Threul et al. by the high inhibitory levels of CSF-2. These high-quality data and attractive model, however, contrast other important studies that clearly illustrated the contribution of monocytes to the renewal of the splenic iron-recycling niche after the stress imposed by damaged RBCs [33,91]. The reason for this discrepancy is not clear. Nevertheless, it may be expected that the defective iron recycling in the spleen may enhance RBCs’ clearance in the liver. Indeed, this seems to be the case in IL-33- and IL1RL1-null mice, characterized by diminished and less phagocytic RPMs and iron deposits in the liver [92]. Interestingly, liver iron levels remained unchanged in *Spic* knock-out mice, raising the question of how iron recycling is compensated in this model. It is likely that the disruption of the heme–SPI-C axis also prevents on-demand RBC clearance in the liver, as *Spic* is also induced during KC differentiation [131]. 

Substantial clinical interest is now focused on the correction of iron status in iron deficiency disorders of different etiologies [113]. The intravenous delivery of new-generation compounds is considered more effective and safer than the oral route. Currently, carbohydrate-coated iron cores are the formulations of choice, and several of these compounds are now FDA-approved drugs, not only to correct iron deficiency but also for diagnostic applications [138]. Interestingly, these iron nanoparticles are targeting mostly tissue and tumor-associated macrophages [138,139]. Therefore, it might be expected that, via the transient iron loading of erythrophagocytosing RPMs and KCs, such drugs may cause the depletion of these cells and hence trigger some degree of iron-recycling niche remodeling. Novel and promising formulations for oral iron delivery that are based on liposomal encapsulation [140] still need to be investigated in more detail for their biodistribution among different cell types. 

Another important context for iron-recycling niche plasticity is the recovery from acute anemia. This process relies on the activation of stress erythropoiesis, which, in mice, is extramedullary and mostly takes place in the spleen. Earlier studies demonstrated that splenic macrophages nourish and support erythrocyte precursors with so-called erythroblastic islands (EBI) and thus are critical for recovery from anemic stress [141,142]. Further studies using a model of bone marrow transplantation and phenylhydrazine-induced hemolysis identified blood monocytes as the major source of early EBI during the recovery process, which next differentiate into RPM-like mature macrophages that further support erythrocyte maturation [143]. Initial monocyte recruitment was linked to the release of the CCL2 chemokine by those resident RPMs that were active in erythrophagocytosis. Follow-up work showed that signaling events in RPMs shape the maturation of the splenic stress erythropoiesis niche [144]. In the early phase, they secrete Wnt ligands to prevent differentiation of the erythroid progenitors and promote their proliferation. In the late phase, when erythropoietin (EPO) levels increase in response to tissue hypoxia, EPO receptor-dependent signaling in RPMs promotes the synthesis of active lipid mediators. Prostaglandin J2 activates intrinsically PPARγ, which suppresses Wnt expression. This de-represses erythroid differentiation, which is additionally stimulated by RPM-derived prostaglandin E2. It remains to be elucidated whether similar PPARγ-mediated signaling events are important for neonatal RPM development that depends on PPARγ [126]. Another important study showed that, during recovery from inflammation-induced anemia, the increased capacity of RPMs for erythrophagocytosis (please see also the chapter devoted to the regulation of erythrophagocytosis) induces *Spic*, which, in turn, triggers the expression of *Gdf15*, one of the cytokines important for erythroid expansion. Taken together, these examples illustrate that the splenic macrophage niche is plastic, responds to external cues and communicates with other cell types to preserve homeostasis. Importantly, erythrophagocytosis intensity acts as an important signaling means to translate environmental conditions to macrophage output behaviors.

Other lines of evidence established further links between inflammatory/danger signals and iron recycling. An important work by Akilesh et al. shed light on the possible etiology of cytopenias in so-called macrophage activation syndrome (MAS), a pathological state that accompanies arthritis and other autoimmune diseases, certain viral infections and malaria [44]. The authors demonstrated that the activation of signaling from TLR receptors 7 and 9 triggers MAS-like syndrome in mice, hallmarked by anemia and thrombocytopenia. This was caused by specialized hemophagocytes that differentiate from monocytes, localized in the spleen, but show a distinct transcriptional signature from RPMs and exhibit higher erythrophagocytic capacity. Others, however, showed that the appearance of hemophagocytes in response to TLR9 ligands, and other signals such as TLR2, TLR3 or TLR4 agonists, as well as TNF-α, IL-6 or IL-17A, is only observed in SIRPα-deficient mice, suggesting a protective role of this “don’t eat me” receptor [145]. Earlier studies demonstrated that specialized hemophagocytes may also arise in response to INFγ signaling and may sequester RBCs via macropinocytosis, a process that is a form of unspecific endocytic fluid-phase engulfment [146]. Pathological hemophagocytosis was also proposed in the etiology of Leishmania-triggered anemia and was linked to SIRPα downregulation [147]. Another report described hemophagocytosis in calcified vascular walls and implicated IL-18 as a signal that promoted this phenomenon [148].

## 8. Cross-Talk between Iron Recycling and Macrophage Immune Functions

Macrophages exhibit a wide spectrum of inflammatory phenotypes, ranging from ‘classically activated’ or M1 macrophages, which are pro-inflammatory, to ‘alternatively activated’ or M2 macrophages, which have immunoregulatory functions [149]. KCs provide anti-inflammatory micromilieu and maintain immune tolerance during homeostasis [150]. Similarly, RPMs were shown to produce immunosuppressive cytokines IL-10 and TGF-β, which promote the differentiation of regulatory T cells [151]. However, recently, RPMs were also implicated in the priming of cytotoxic T lymphocytes during an antiviral immune response [152]. Early work showed that transfusion of stored RBCs induces inflammation and favors bacteria growth, likely due to the higher iron availability for the pathogens [153]. In line with these findings, it has been reported that excessive iron loading and heme exposure polarize macrophages in the liver and spleen into a pro-inflammatory M1 phenotype [154]. Interestingly, two recent pieces of work illustrated that intensified erythrophagocytosis in the liver provokes immunosuppressive rather than immunostimulatory skewing of myeloid cells. Olonisakin et al. showed that transfusion of stressed RBCs before *Klebsiella pneumoniae* infection promotes bacteria growth and increases the risk of fatal sepsis [155]. This phenomenon was shown to be independent of the iron acquisition by bacteria but was mediated by the weakened antibacterial immune response. Whole liver transcriptional profiling indicated that the immunosuppressive effects of forced RBC degradation were mediated by the impairment of STAT1 signaling. Further in vitro studies identified the heme protoporphyrin ring but not iron as the hemoglobin-derived entity that is responsible for the compromised inflammatory response and showed that it acted via NRF1 and NRF2 signaling. Consistently, another study, which employed single-cell RNA sequencing, demonstrated clearly that an increased burden of defective RBCs leads to the appearance of strongly immunosuppressive myeloid cells in the liver [32]. Of clinical significance, their appearance was shown to be protective in two models of macrophage-driven hepatitis. Interestingly, the authors reported that, in vitro, heme-polarized macrophages exhibited a unique transcriptional signature, distinct from M1 or M2 macrophages. The enhanced rate of RBC engulfment was also recently linked to an increased risk of sepsis in another pathophysiological setting [16]. It was reported that the increased mucosal permeability of the intestine, characteristic of, e.g., inflammatory bowel disease, and the consequent transfer of bacterial components from the gut to the bloodstream impair the synthesis of unsaturated fatty acids in the liver. This, in turn, results in the decreased membrane fluidity of RBCs that triggers their premature clearance by splenic RPMs. Such an enhanced rate of erythrophagocytosis was proposed to underlie the elevation of body iron levels that promoted bacteria growth.

Investigations of tumor-associated macrophages (TAMs), cells that are known as strongly immunosuppressive, have revealed another layer of complexity regarding the cross-talk between iron recycling and immune polarization. It was demonstrated that a subset of TAMs that are located in the hemorrhagic tumor areas and become iron-loaded show a pro-inflammatory profile and enhanced anti-tumor activity [156]. Delivery of iron nanoparticles to TAMs was proven to be a promising therapeutic approach to enhance tumor immune eradication. However, interestingly, other lines of evidence showed that the deletion of HO-1 in TAMs, which is expected to decrease the iron release following the engulfment of RBC components, boosts anti-tumor immunity [157]. Likewise, genetic depletion of a subset of CD163-positive TAMs, likely capable of Hb uptake, improves immune responses against cancer cells [158,159]. This is also consistent with the observation that anti-inflammatory agents, glucocorticoids, strongly induce CD163 expression [160].

Taken together, it becomes apparent that iron-recycling myeloid cells are highly heterogeneous and respond to disturbances of heme and iron balance, or exposure to stressed/senescent/hemolytic RBCs, in a context-dependent manner. More work is required to better understand how iron management in different subsets of macrophages may alter their immune functions. 

## 9. Concluding Remarks

The growing body of work sheds light on new, exciting aspects of iron recycling. However, many questions remain open. The mechanistic details of RBC recognition, phagocytosis, degradation and the further processing of heme-derived iron are incompletely understood, and we lack knowledge of whether these processes are distinct between RPMs and KCs. It is not known if other cell types than those of myeloid origin contribute to RBC clearance and iron turnover in physiological or pathological conditions. Finally, we are only starting to identify how erytrophagocytosis is linked to other processes within or outside the tissue microenvironment of iron-recycling macrophages. We expect that many of these still elusive facets of iron turnover might be uncovered by future studies.

## Data Availability

No new data were created or analyzed in this study. Data sharing is not applicable to this article.
